# Recurrent c.G1636A (p.G546S) mutation of *COL2A1* in a Chinese family with skeletal dysplasia and different metaphyseal changes: a case report

**DOI:** 10.1186/s12887-017-0930-9

**Published:** 2017-07-24

**Authors:** Jing Chen, Xiaomin Ma, Yulin Zhou, Guimei Li, Qiwei Guo

**Affiliations:** 1 United Diagnostic and Research Center for Clinical Genetics, School of Public Health of Xiamen University & Xiamen Maternal and Child Health Hospital, Xiamen, Fujian China; 2 Department of Child Health, Maternal and Child Health Hospital, Xiamen, Fujian China; 3 Department of Radiology, Maternal and Child Health Care Hospital, Xiamen, Fujian China; 4 Department of Pediatrics, Shandong Provincial Hospital Affiliated to Shandong University, Jinan, China

**Keywords:** c.G1636A, p.G546S, *COL2A1*, Dappling, Corner fracture

## Abstract

**Background:**

Mutations in the *COL2A1* gene cause type II collagenopathies characterized by skeletal dysplasia with a wide spectrum of phenotypic severity. Most *COL2A1* mutations located in the triple-helical region, and the glycine to bulky amino acid substitutions (e.g., glycine to serine) in the Gly-X-Y repeat were identified frequently. However, the same *COL2A1* mutations are associated with different phenotypes and the genotype-phenotype relationship is still poorly understood. Therefore, the studies of more patients about the recurrent mutations in *COL2A1* will be needed for further research to provide more comprehensive clinical and genetic data. In this paper, we report a rare recurrent c.G1636A (p.G546S) mutation in *COL2A1* associated with different metaphyseal changes in a Chinese family.

**Case presentation:**

The proband (III-3) was the second child of the family with skeletal dysplasia. She was 2 years and 3 months old with disproportional short stature, short neck, pectus carinatum, genu varum, bilateral pes planus, and obvious waddling gait. Notably, she displayed severe metaphyseal lesions, especially typical “dappling” and “corner fracture” appearance, whereas no particular metaphyseal involvement was detected in the proband’s mother (II-3) and elder sister (III-2) in the family. We identified a heterozygous mutation (c.1636G > A) in *COL2A1* in the three patients, causing the substitution of glycine to serine in codon 546. Although the same mutation has been reported in two previous studies, the phenotypes of the previous patients were different from those of our patients, and the characteristic “dappling” and “corner fracture” metaphyseal abnormalities were not reported previously.

**Conclusions:**

In this study, we identified a c.G1636A (p.G546S) mutation in the *COL2A1* associated with different metaphyseal changes, which was never reported in the literature. Our findings revealed a different causative amino acid substitution (glycine to serine) associated with the “dappling” and “corner fracture” metaphyseal abnormalities, and may provide a useful reference for evaluating the phenotypic spectrum and variability of type II collagenopathies.

## Background

The type II collagen gene (*COL2A1*, MIM #108300) encodes the alpha 1(II) chain of procollagen type II, which is crucial for constructing functional collagen. Mutations in this gene cause type II collagenopathies, which are skeletal dysplasias with a wide spectrum of phenotypic severity [1]. The most severe phenotypes include achondrogenesis type II and hypochondrogenesis, which are associated with neonatal death [2]; the intermediately severe phenotypes, such as spondyloepiphyseal dysplasia congenita (SEDC) [3] and spondyloepimetaphyseal dysplasia (SEMD), Strudwick type [4], are associated with disproportionately short stature, abnormal epiphyses, scoliosis, and/or ocular conditions; and the mildest phenotypes, such as osteoarthritis [5] and stickler syndrome type I [6] manifesting only in late childhood or adulthood, and present as isolated joint or ocular disease.

According to the Leiden Open Variation Database (LOVD, http://databases.lovd.nl/shared /genes/*COL2A1*), 455 variations in *COL2A1* have been reported (updated on March 24, 2016). Due to the rarity of recurrent mutations, no mutational hot spots have been identified. Type II collagen is a homotrimer composed of three alpha1 (II) chains. Each alpha 1 (II) chain contains a triple-helical structure formed by a characteristic Gly-X-Y repeat sequence. The X and Y position of the Gly-X-Y repeat are occupied by proline and hydroxyproline residues, respectively [7]. Most *COL2A1* mutations are located in the triple-helical region, and glycine to bulky amino acid substitutions (e.g., glycine to serine) in the Gly-X-Y repeat have been identified frequently [8], however, the same *COL2A1* mutation may cause different phenotypes and the genotype- phenotype relationship is still poorly understood. In this study, we identified a recurrent c.G1636A (p.G546S) *COL2A1* mutation in a Chinese family. The clinical phenotypes of three affected family members were described. This mutation is associated with a specific spondyloepimetaphyseal dysplasia characterized by “dappling” and “corner fracture” metaphyseal abnormalities in one of the three family members with skeletal dysplasia, which was never reported in the previous literature.

## Case presentation

**Fig. 1:**
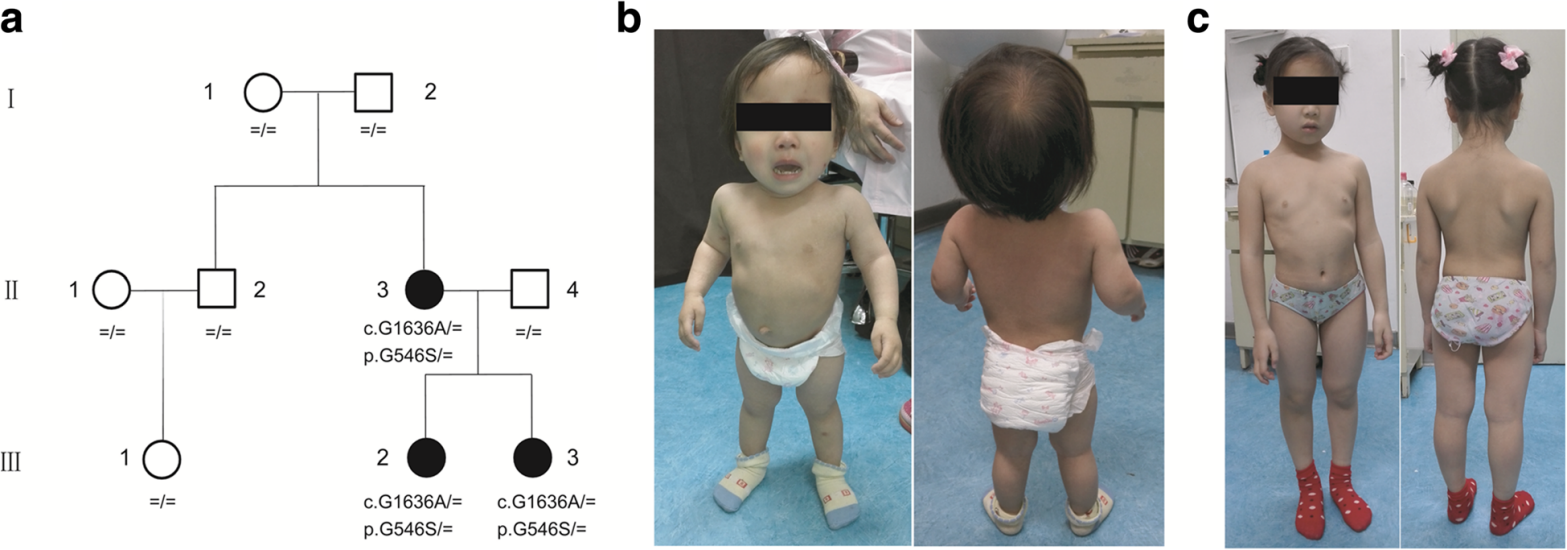
Pedigree and pictures of the patients. **a** Pedigree of the patients. **b** Pictures of patient III-3. **c** Pictures of patient III-2

The pedigree of the patients is shown in Fig. 1a. The proband (III-3) was the second child in the family with skeletal dysplasia. She was born at 40^+3^ weeks of gestation by cesarean. Her birth length and weight were reported to be 46.0 cm (<3rd centile) and 2700 g (3rd–10th centile), respectively. She was brought to the Department of Pediatrics at the age of 2 years and 3 months for disproportional short stature. Her height was 66.5 cm (<3rd centile); her weight was 8.0 kg (<3rd centile); and her head circumference was 48.2 cm (50th–75th centile). Other physical examination findings included short neck, pectus carinatum, genu varum, bilateral pes planus, and an obvious waddling gait (Fig. 1b). Her early motor development was slightly delayed, while her intellectual development was normal. In contrast, the proband’s elder sister (III-2) displayed milder symptoms: she was born at 40^+5^ weeks of gestation by cesarean. Her birth length and weight were reported to be 48.0 cm (10th–25th centile) and 2800 g (10th–25th centile), respectively. She was brought to our clinic at the age of 8 years and 7 months. Her height was 108.5 cm (<3rd centile), and her weight was 21.0 kg (3rd–10th centile). Besides short stature, no remarkable abnormalities were found in the physical examination (Fig. 1c). The proband’s mother (II-3) was 33 years old when she received the physical examination. Her height was 128.5 cm (<3rd centile), and her weight was 35.2 kg (<3rd centile). Similar to her first child, no remarkable abnormalities were found except for the short stature. Unfortunately, she did not consent to taking pictures of her profile. None of the three patients displayed ocular defects, hearing impairment, inguinal hernia, or cleft palate.

**Fig. 2:**
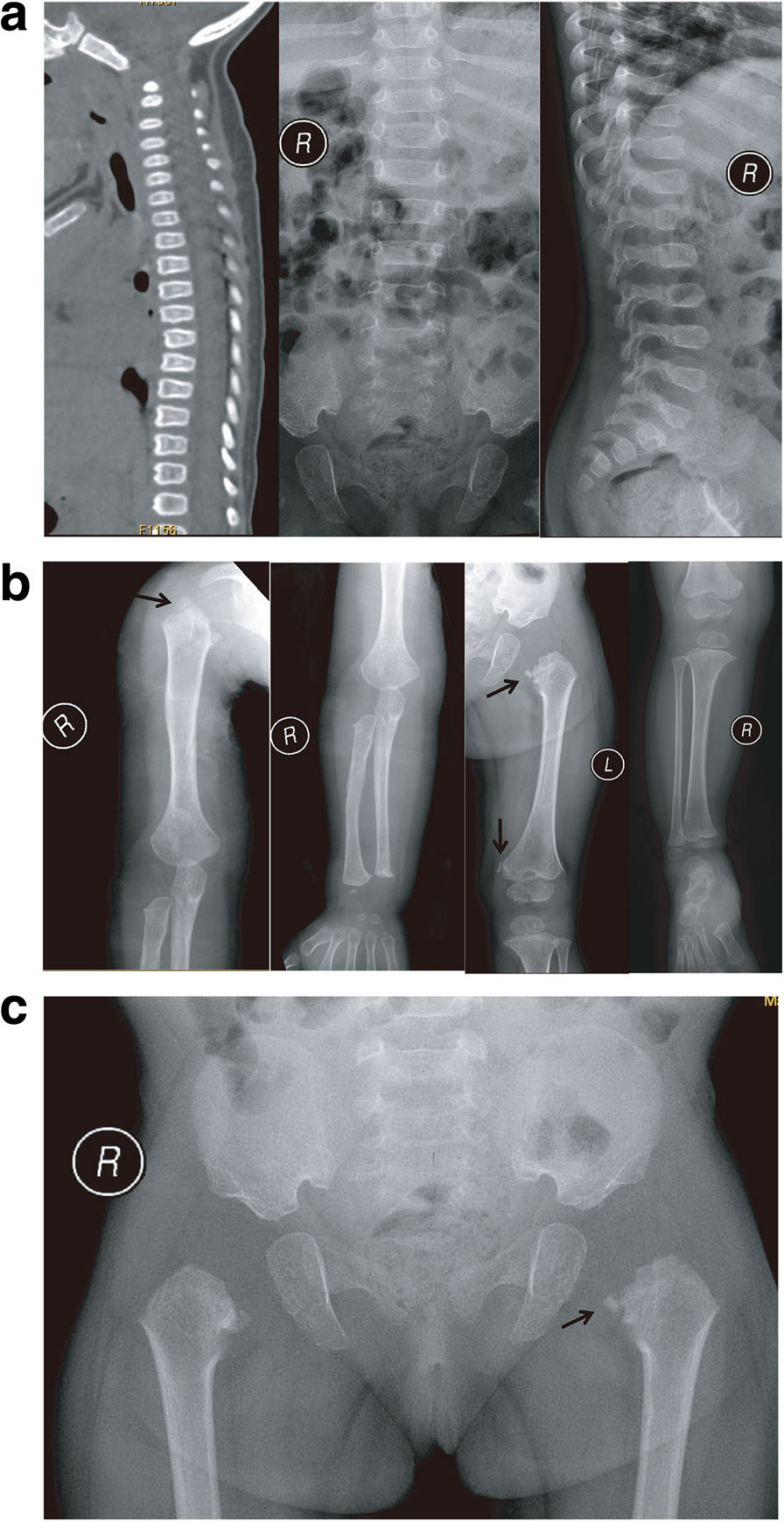
Radiographic findings of patient III-3. **a** Radiographic findings of the spine of patient III-3. The patient displayed platyspondyly (C3–C7), defects on the edge of the anterior vertebral bodies (L3–L5), and a slight shift of the vertebral axis. In addition, ovoid vertebral bodies, which are indicators of dysplasia, were observed in the CT images of the cervical spine. **b** Radiographic findings of the long bones of patient III-3. Bilateral humeri, ulnae, radii, femurs, and tibiofibulas were shortened. Bilateral femoral heads and necks, as well as the femoral head epiphyses and distal humeral epiphyses, were absent. The epiphyses of the upper humeri and distal tibias were dysplastic. The metaphyses of the proximal femurs and proximal humeri displayed a “dappling” appearance, resulting from the irregular intermingling of radiolucencies and radiodensities. The metaphyses in the proximal tibias were flared and irregular. Notably, “corner fracture” phenomena were observed in the right proximal humerus and bilateral femurs (arrows). **c** Radiographic findings of the pelvis of patient III-3. An irregular acetabular roof was observed

**Fig. 3:**
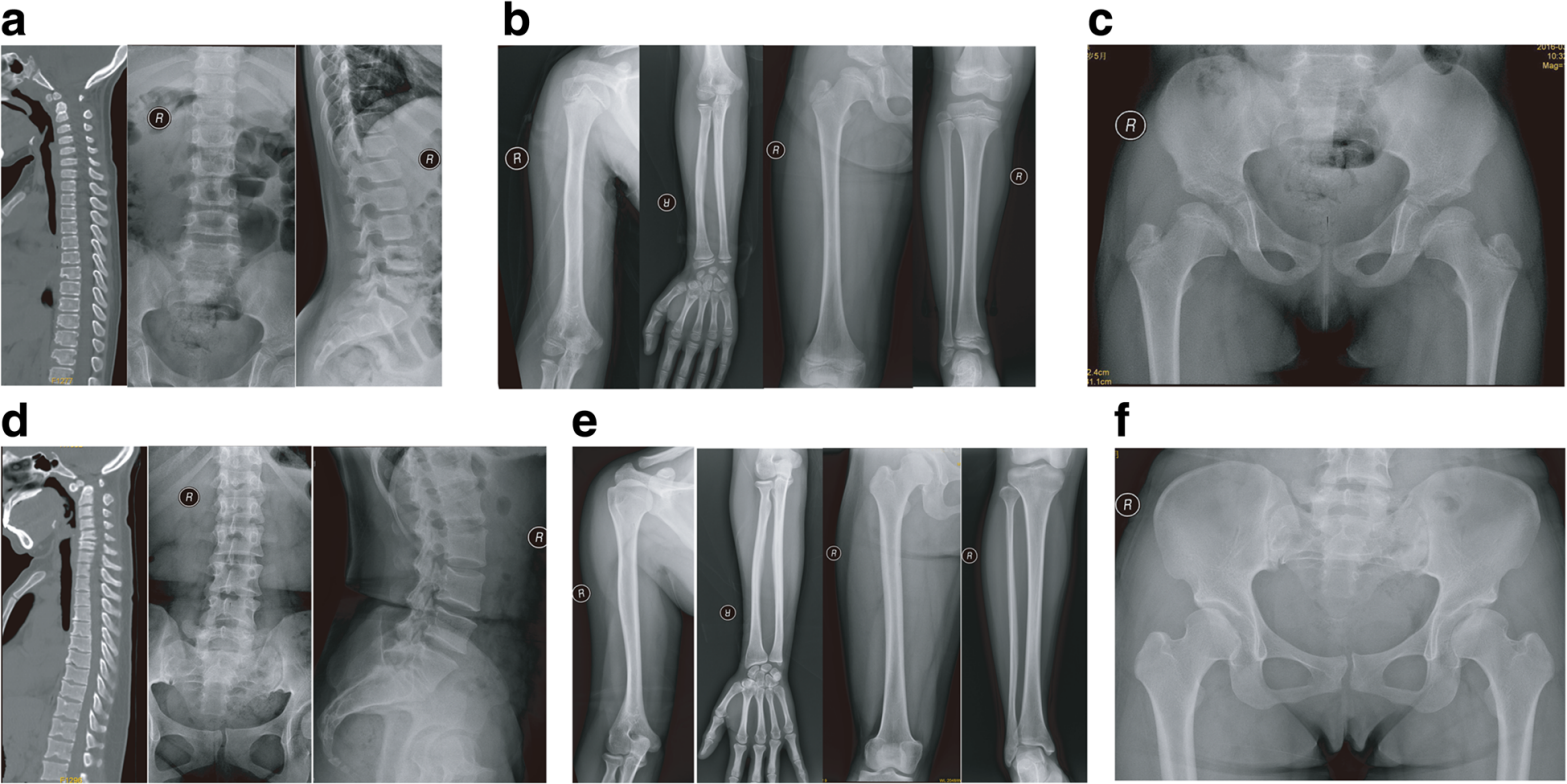
Radiographic findings of patients III-2 and II-3. **a** Radiographic findings of the spine of patient III-2. The patient displayed platyspondyly (C3–C6) and defective anterior vertebral bodies (T7–T12, particularly at the lower edge). **b** Radiographic findings of the long bones of patient III-2. Dysplasia was detected in the bilateral femoral heads and distal tibial epiphysis. No particular changes were found in the metaphyses. **c** Radiographic findings of the pelvis of patient III-2. No particular abnormalities were found in the pelvis. **d** Radiographic findings of the spine of patient II-3. Os odontoideum and atlantoaxial subluxation were observed in the CT scan of the cervical spine. Other findings included multiple Schmorl’s nodes (T5–T12), platyspondyly (C5–C6, T6–T9), lumbar lordosis, and a marked increase in the lumbosacral angle. **e** Radiographic findings of the long bones of patient II-3. Dysplasia was found in the bilateral femoral heads and distal tibial epiphysis. No particular changes were found in the metaphyses. **f** Radiographic findings of the pelvis of patient II-3. No particular abnormalities were found in the pelvis

Radiographic examinations were performed on the three patients (Figs. 2 and 3). In general, the skeletal defects of patients II-3 and III-2 were milder than those of patient III-3. For patients II-3 and III-2, the major affected structures were the spine and epiphyses, whereas in patient III-3, skeletal defects were found in the spine, epiphyses, and pelvis. Notably, patient III-3 displayed severe metaphyseal lesions, especially a typical “dappling” and “corner fracture” appearance. In contrast, no particular metaphyseal involvement was detected in patients II-3 and III-2.

Written informed consent was obtained from the patients (or guardian) and their family members for conducting the genetic tests and publishing the research data. The study protocol was approved by the ethics committee of Xiamen Maternal and Child Health Hospital. We collected peripheral blood samples from three generations of the patients’ family (Fig. 1a). Genomic DNA was extracted from 200 μL of blood using the Super/HF16 plus DNA Extraction System (MagCore, Xiamen, China) according to the manufacturer’s protocol. DNA samples from the three patients (II-3, III-2, and III-3) were analyzed by commercial whole exome sequencing (WES; Sinopath, Beijing, China). A guanine to adenosine change at position 1636 of the coding sequence of the *COL2A1* gene (c.G1636A), leading to a corresponding glycine to serine change in the protein sequence (p.G546S), was detected in all of three patients by WES. The mutation was confirmed by Sanger sequencing. Related family members were also examined for this mutation by Sanger sequencing. The sequencing results revealed that the mutation found in patient II-3 was a de novo mutant because it was absent in the genomes of her parents (I-1 and I-2). In addition, a total of 15,116 variants were unique in the exome of patient III-3 compared to in patients II-3 and III-2, including three heterozygous variants in *COL2A1* (Table 1).

**Table 1:** *COL2A1* variants in the exome data of patient III-3

Variant	Nucleotide change	Protein change	^a^Functional prediction by SIFT database	^b^Functional prediction by PolyPhen2 database	^c^Conservative alignment between species using HomoloGene database
rs140740708	c.2854G > T	p.P952T	Tolerated	Benign	Conserved
rs1635560	c.4317 + 43G > A	-	-	-	-
rs41272029	c.2673G > C	p.G891G	-	-	Highly conserved

^a^SIFT database (http://sift.jcvi.org/)

^b^Polyphen2 database (http://genetics.bwh.harvard.edu/pph2/)

^c^HomoloGene database (http://www.ncbi.nlm.nih.gov/homologene)

Eventually, based on previous studies and the current classification of skeletal dysplasia [9–13], patient III-3 was diagnosed with a variant of SEMD, Strudwick type, and patients II-3 and III-2 were diagnosed with mild SEDC.

## Discussion

In the differential diagnosis, the “dappling” metaphyseal appearance, which results from irregular ossification, is characteristic of SEMD, Strudwick type (MIM #184250), while the “corner fracture” metaphyseal appearance, which was considered as an extra ossification center, is characteristic of spondylometaphyseal dysplasia, corner fracture type (MIM #184255). Thus far, four publications have reported a phenotype similar to that of patient III-3, with a combination of “dappling” and “corner fracture” metaphyseal abnormalities, and *COL2A1* mutations were also detected in the patient in those studies [9–12]. Including our patient, a total of six patients of different gender and race have been described who display similar phenotypes, including the characteristic “dappling” and “corner fracture” metaphyseal abnormalities, disproportional short stature, relatively mild abnormalities in the spine with platyspondyly, shortened long bones with relatively normal small tubular bones in the hands and feet, dysplasia of the femoral heads and necks, hip dysplasia, and genu varum/valgum (Table 2). According to previous studies and the current classification of skeletal dysplasia [9–13], this distinct phenotype was classified as a variant of SEMD, Strudwick type. An interesting finding from these studies is that most *COL2A1* mutations associated with the “dappling” and “corner fracture” metaphyseal abnormalities were glycine to arginine substitutions (in four of six patients), which suggests a potential molecular mechanism. Although more patients are needed to delineate a possible molecular mechanism, our patient reveals a different causative amino acid substitution (glycine to serine), which expands the mutational spectrum of this specific phenotype. We anticipate more patients will be discovered, which will further delineate and decipher this specific variant of SEMD, Strudwick type.

**Table 2:** Phenotypic comparison of the six patients with “dappling” and “corner fracture” metaphyseal abnormalities

	Patient 1 [Kaitila and others 1996] [9]	Patient 2 [Kaitila and others 1996] [9]	Patient 3 [Walter and others 2007] [12]	Patient 4 [Walter and others 2007] [12]	Patient 5 [Matsubayashi and others 2013] [10]	Patient 6 [Our study]	SEMD- Strudwick type
Mutation Gender Nationality Physical examination	Gly154Arg male Finnish	Gly154Arg female unknown	Gly181Arg female unknown	Gly922Arg female unknown	Gly861Val male Japanese	Gly546Ser female Chinese	
Disproportional short stature	+	+	+	+	+	+	+
Spinal deformity							
Scoliosis	−	−	+	−	−	−	+
Kyphosis	+	−			−	−	+
Lumbar lordosis	+	+	+	−	+	−	+
Chest deformity							
Pectus excavatum	+	−	−	−	unknown	+	−
Pectus carinatum	−	−	+	+	unknown	+	+
Limbs							
Short	+	+	+	+	+	+	+
Genu varum/valgum	+	+	+	+	+	+	+
Normal mentation	+	+	+	+	+	+	+
Inguinal hernia	−	−	unknown	unknown	unknown	−	+
Cleft palate	−	−	−	−	−	−	−
Myopia	−	+	−	−	+	−	+
Retinal detachment	−	−	unknown	unknown	unknown	−	+
Hearing loss	−	+	−	−	+	−	−
Radiographic findings							
Platyspondyly	+	+	+	+	+	+	+
Odontoid hypoplasia	+	+	−	+	unknown	−	+
Flaring and irregularities of metaphyses	+	+	+	+	+	+	+
“Corner fracture” appearance of metaphyses	+	+	+	+	+	+	−
“Dappling” appearance of metaphyses	+	+	+	+	+	+	+
Epiphyseal dysplasia	+	+	+	+	+	+	+
Shortened long bones	+	+	+	+	+	+	+
Normal small tubular bones	+	+	+	+	+	+	+
Dysplasia of femoral heads and necks	+	+	+	+	+	+	+
Hip dysplasia	+	+	+	+	+	+	+
Autosomal dominant	+	+	+	+	+	+	+

Currently, the genotype-phenotype correlations in type II collagenopathies cannot be clarified for several reasons [14, 15]. First, there are no mutational hot spots, and most mutations are unique. Second, there is a wide range of phenotypic variation among patients, even in individuals who share the same mutation. Moreover, age-dependent transitions and/or other unidentified factors could also complicate the clinical phenotypes. However, the study of recurrent *COL2A1* mutations provides an opportunity to gain insight into the phenotypic spectrum and variability of individual mutations or mutation groups, which could facilitate a more precise prognosis early in life, thus improving individualized medical care and patients’ quality of life.

Recurrent *COL2A1* mutations have been reported in several studies; some mutations displayed similar phenotypes, while others displayed distinct phenotypes [8, 15–20]. For example, Silveira et al. reported clinical and radiological follow-up of six unrelated patients with a R989C mutation that was associated with a severe SEDC phenotype, which was consistent with the phenotypes of twelve other R989C mutation cases [18]. In contrast, three patients with a G504S mutation showed mild SEDC, SEDT, and severe SEDC phenotypes [8, 15]. Likewise, a G513S mutation in a 4-year-old was associated with mild SEDC, but was also associated with a lethal form of SEDC that resulted in neonatal death [15, 19]. Based on previous limited data, unlike glycine to non-serine substitutions, glycine to serine substitutions produced variable effects, with both inter- and intra-familial phenotypic variation [8, 15].

Two previous reports of the c.G1636A (p.G546S) mutation were found in the online database. Xu et al. reported the c.G1636A mutation in a familial case of SEDC [20]. Unlike our patients, the major skeletal abnormalities in Xu et al.’s patients were concordant among affected family members and included dysplasia of the femoral heads and necks, abnormal acetabular roofs, moderate or mild scoliosis, and thoracic hyperkyphosis. Most of these skeletal abnormalities were not found in our patients, except for dysplasia of the femoral heads and necks and abnormal acetabular roofs, which were observed in patient III-3. In addition, marked metaphyseal abnormalities were noted in one of our patients (III-3), which was distinct from the phenotypes of Xu et al.’s patients. Kaissi et al. reported another patient of a c.G1636A mutation in a patient in Germany [21]. As the authors stated in the English abstract, the patient was characterized by short stature associated with acetabulo femoral dysplasia, spinal osteochondritis (Scheuermann’s disease), and mild thoracic kyphosis. According to the limited phenotypic information, the skeletal abnormalities in this patient were similar to those observed in Xu et al.’s patients. Therefore, in agreement with the previous findings for glycine to serine substitutions [8, 15], in this study, patients with the G546S mutation show inter- and intra-familial phenotypic variation. Due to the small number of patients with insufficient genetic information and the complicated genotype-phenotype correlation, the reason why the same *COL2A1* mutation causes different phenotypes is still unclear. A reasonable hypothesis is that in addition to the causative *COL2A1* mutation in a critical domain, other genetic, epigenetic, and environmental factors can be attributed to inter- and intra-familial phenotypic variation by influencing the microenvironments within the collagen domains or complex interactions with other proteins [22]. In our WES data, numerous variants were found to be unique in the exome of patient III-3 compared to in the other two patients, particularly two variants in *COL2A1*: one was a benign c.2854 C > A (p.P952T) located outside the triple helix repeat domain while the other was a c.4317 + 43G > A variation located in the intron region (Table 1). These data provide potential candidates for gaining insight into the phenotypic spectrum and variability of type II collagenopathies. However, the contribution of these genetic variations should be further investigated in a larger number of clinical samples and functional studies using genetic animal models. The use of genome-wide strategies, e.g., genome-wide association study, whole genome/exome sequencing, and whole genome bisulfate sequencing, with large cohorts of patients may reveal the basis of the indefinite genotype-phenotype correlation of *COL2A1*.

## Conclusion

Our case reported a recurrent c.G1636A (p.G546S) mutation of *COL2A1* in a Chinese family with skeletal dysplasia. Specific spondyloepimetaphyseal dysplasia characterized by “dappling” and “corner fracture” metaphyseal abnormalities was observed in one of the three family members. Our finding revealed a different causative amino acid substitution (glycine to serine) associated with the “dappling” and “corner fracture” metaphyseal abnormalities, and may provide a useful reference for evaluating the phenotypic spectrum and variability of type II collagenopathies.

## Abbreviations

SEDC: Spondyloepiphyseal dysplasia congenital
SEMD: Spondyloepimetaphyseal dysplasia
WES: Whole exome sequencing
